# Distribution Characteristics of Microplastics in Surface Seawater off the Yangtze River Estuary Section and Analysis of Ecological Risk Assessment

**DOI:** 10.3390/toxics11110889

**Published:** 2023-10-30

**Authors:** Xiao Ji, Shuaishuai Yan, Yanlong He, Haisheng He, Hanqi Liu

**Affiliations:** 1 East China Sea Ecological Center, MNR (Ministry of Natural Resources), Shanghai 201206, China; jixiao@ecs.mnr.gov.cn (X.J.); yanshuaishuai@ecs.mnr.gov.cn (S.Y.); ylhe@ecs.mnr.gov.cn (Y.H.); hehaisheng2018@163.com (H.H.); 2 Key Laboratory of Marine Ecological Monitoring and Restoration Technology, Ministry of Natural Resources, Shanghai 201206, China

**Keywords:** microplastic, surface seawater, Yangtze River estuary

## Abstract

Microplastics are widespread in the oceans as a new type of pollutant. Due to the special geographical environment characteristics, the Yangtze River estuary region become hotspot for microplastics research. In 2017 and 2019, surface seawater microplastics samples were collected from five stations off the Yangtze River estuary during four seasons (spring, summer, autumn, and winter). The abundance and characteristics of microplastics in seawater were researched. The results showed that microplastics widely existed in surface seawater; the average abundance of microplastics in seawater was (0.17 ± 0.14) items/m^3^ (0.00561 ± 0.00462) mg/m^3^; and accounting for 80% of the total plastic debris, the abundance of microplastics was at moderately low levels compared to national and international studies. The particle size of most microplastics was between 1 mm to 2 mm, accounting for 36.1% of the total microplastics. The main shapes of microplastics were fiber, flake, and line, accounting for 39.5%, 28.4%, and 20.8%, respectively. Polypropylene, polyethylene terephthalate, and polyethylene were the main components of microplastics, accounting for 41.0%, 25.1%, and 24.9%, respectively. Yellow, green, black, and transparent were the most common colors, accounting for 21.9%, 19.6%, 16.5%, and 15.7%, respectively. This study shows that the spatial distribution of microplastics in the surface waters off the Yangtze River estuary shows a decreasing trend from nearshore to farshore due to the influence of land-based inputs, hydrodynamics, and human activities; the distribution of microplastics has obvious seasonal changes, and the level of microplastic pollution is higher in summer. The potential ecological risk of microplastics in the surface waters off the Yangtze River estuary is relatively small.

## 1. Introduction

The concept of microplastics was first proposed by Thompson [1] in 2004. Seawater microplastics are plastic debris including fibers, particles, and fragments less than 5 mm in length that are present in the marine environment and mainly formed through the degradation of large plastic fragments by mechanical and photo-oxidation pathways [2,3,4]. These include primary and secondary microplastics [5]. Because microplastics are chemically stable and can exist in the environment for hundreds to thousands of years [6], they have received increasing attention as a new class of environmental pollutants. Microplastic pollution has become a global environmental problem that is widespread in the terrestrial and marine environments. Traces of microplastics have been found from land [7] to ocean [8,9,10], from nearshore [11,12] to pelagic to deep sea [13], from plateau lakes [14] to polar regions [15,16], from *Aeromonas* sp. [17,18] to marine mammals [19,20], and then to food [21] and cosmetics [22], which are closely related to human exposure.

Seawater microplastics have become a research hotspot for environmental issues in recent years, and scholars both domestic and abroad have carried out extensive studies, most of which focused on nearshore estuaries, bays, and other areas with intensive human activities. Scholars in China have carried out relevant studies in the Yangtze River estuary, South China Sea, Bohai Bay, etc., and the basic abundance ranges from 0.045 to 8.91 items/m^3^. Microplastics in the surface waters of different seas in other regions of the world [23] have abundance ranges from 0.19 to 7.68 items/m^3^. However, there are still fewer studies related to seasonal abundance changes.

Studies have mainly focused on offshore areas and beach surveys [24,25,26,27,28,29,30]. Therefore, in this paper, the seasonal distribution and compositional characteristics of microplastics in the outer surface seawater of the Yangtze River estuary were studied with the neighboring waters of the Yangtze River estuary as the study area to reveal the distribution characteristics of this microplastic and provide data support for the implementation of source control and management of microplastic pollution.

## 2. Materials and Methods

### 2.1. Study Area

**Figure 1:**
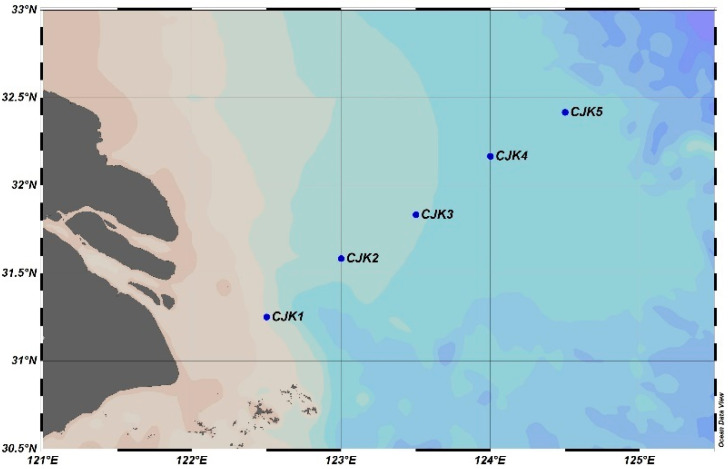
Sampling sites.

The study area is located in the sea area off the Yangtze River estuary, with five stations (31.25°~32.42° N, 122.5°~124.5° E), and the section direction was laid out to coincide with the direction of the freshwater flushing in the Yangtze River estuary. Sampling was carried out in May 2017 (spring), November 2017 (autumn), February 2019 (winter), and August 2019 (summer), and the sampling stations are shown in Figure 1.

### 2.2. Sample Collection

In this study, a manta net was used to collect floating microplastics in surface seawater. The mouth of the net is 1 m long and 0.5 m wide; the net coat is a biological sampling net made of silk (the main component is protein), which is 3 m long, with an aperture of 330 μm, and a stainless-steel net bottom tube is connected at the bottom. There is a fixed flow meter in the center of the net mouth for calculating the amount of excess water. The vessel traveled at a speed of 2–3 knots, and each tow lasted 10–15 min. Before and after each tow, the net was rinsed, and the samples in the bottom tube were transferred to glass vials to avoid interfering with the samples between stations for further analysis in the laboratory.

### 2.3. Laboratory Analysis

The samples were passed through 5 mm and 330 µm stainless-steel mesh sieves sequentially, impurities such as fish and shrimp were rinsed and discarded, and the retained material from the 330 µm mesh sieve was rinsed with purified water and placed into a 500 mL beaker. Plastic samples of more than 5 mm were rinsed and stored separately for further analysis. The beakers and samples were dried in an oven at 60 °C. After drying, 20 mL of 0.05 mol/L ferrous sulfate solution and 20 mL of 30% hydrogen peroxide were added sequentially and digested at room temperature. If organic matter was still seen after digestion, we continued to add hydrogen peroxide and repeated the above operation until the organic matter in the sample was completely dissolved. After complete digestion, 6 g of NaCl solid was added to each 20 mL of digestion solution, dissolved, transferred to a glass funnel sealed at the lower end with a stopcock, and allowed to stand for density separation. The lower impurities were transferred to a beaker, and the supernatant was filtered using a glass fiber filter membrane (Whatman GF/F, 47 mm in diameter with a pore size of 0.7 μm), which was placed in a Petri dish and dried at 60 °C for further analysis.

The samples were observed using a stereo microscope to select the suspected plastic fragments, particles, etc., which were photographed using a stereo microscope with a photo-camera system (Nikon SMZ25, Nikon Corporation, Yokohama, Japan), and information on the physical characteristics of the plastics, such as shape, color, and size, was observed and documented using software (NIS-Elements D 4.50.00, Nikon Corporation, Yokohama, Japan) that was used in conjunction with the camera system. We used imageJ (1.5) software to first record the scale of the photographic process and later calculated the size of the microplastic within a fixed pixel by converting between pixel and scale. The chemical composition of the samples was analyzed using a Fourier transform infrared microspectrometer (Nicolet iN 10 MX, Thermo Fisher, Waltham, MA, USA) transmission mode-MCT detector.

### 2.4. Contamination Mitigation

To prevent the samples from being contaminated by plastic fibers in the environment, cotton coats were worn during the analyses, and the surfaces of the operation platforms were wiped. All the glassware was thoroughly washed and covered with aluminum foil, and all prepared solutions were filtered through membranes before being used. The Petri dishes, filter membranes, and tweezers were inspected under a microscope to ensure that there was no microplastic pollution. When the laboratory digestion was conducted, an experimental blank was evaluated at the same time. The value of the blank was deducted when the results were calculated.

### 2.5. Statistical Analysis

The distribution of microplastics was mapped using the software Ocean Data View (5.1.5) and statistically analyzed and tabulated using Microsoft Office Excel 2016. The significant difference were measured via nonparametric tests (Mann–Whitney U-test). Moreover, we provide concentration methods for both the individual and mass benchmarks. The average density of microplastics used for the conversion in the paper was derived from Zhao [31].

## 3. Results

### 3.1. Seasonal Abundance Distribution of Microplastics

**Figure 2:**
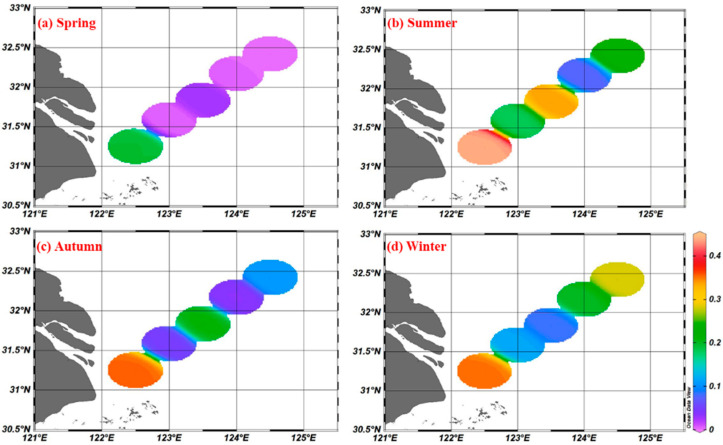
Distribution of microplastics in surface seawater of different season (items/m^3^).

In this study, four surveys were carried out in the area off the Yangtze River estuary, and a total of 1825 plastic samples (with a size larger than 5 mm) and 1455 microplastic samples (with a size smaller than 5 mm) were analyzed, and microplastics accounted for 80% of the total. The average abundance of microplastics in the surface seawater off the Yangtze River estuary was (0.17 ± 0.14) items/m^3^ (0.00561 ± 0.00462) mg/m^3^. The average concentrations of microplastics in the four seasons of spring, summer, autumn, and winter were (0.05 ± 0.08) items/m^3^ and (0.00165 ± 0.00264) mg/m^3^, (0.25 ± 0.14) items/m^3^ and (0.00825 ± 0.00462) mg/m^3^, and (0.15 ± 0.13) items/m^3^ (0.00495 ± 0.00429) mg/m^3^ and (0.21 ± 0.11) items/m^3^ (0.00693 ± 0.00363) mg/m^3^, respectively. The concentrations of microplastics in the summer were higher than that in other seasons, and the abundance of microplastics in different seasons was in the same order of magnitude (Figure 2). The spatial distribution of microplastics shows that the nearshore abundance is higher than the farshore abundance in the sea area off the Yangtze River estuary.

### 3.2. Particle Size, Shape, Color, and Composition of Microplastics

**Figure 3:**
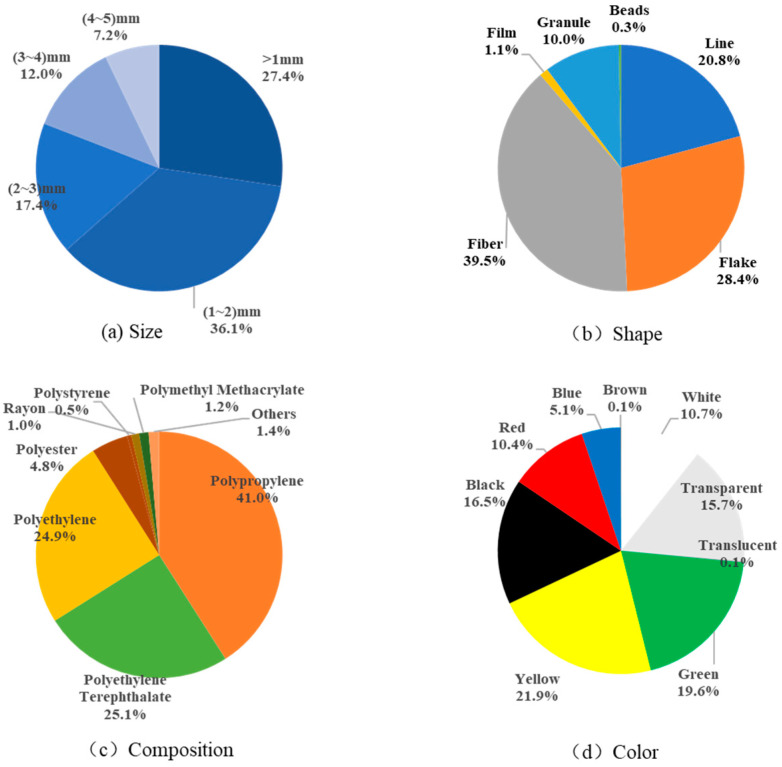
Physical and chemical characteristics of microplastics in the surface seawater off the Yangtze River estuary.

**Figure 4:**
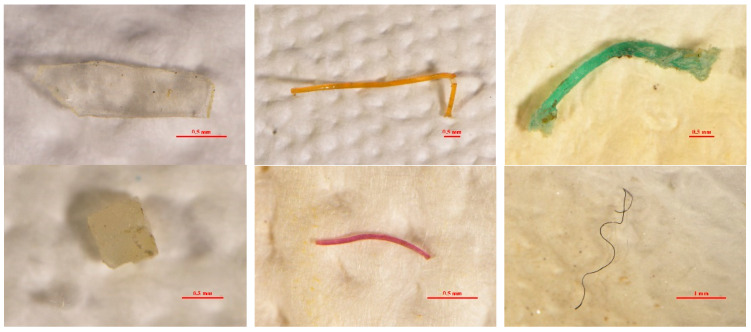
The types of microplastics in the surface seawater.

The physicochemical characteristics of microplastics in the surface seawater off the Yangtze River estuary are shown in Figure 3 and Figure 4. The results show that microplastics with particle size in the range of (1–2) mm are the most numerous, accounting for 36.1% of all microplastics; the number of microplastics tends to increase with the decrease in particle size; and plastics tend to be miniaturized in the ocean, which is consistent with other studies [32,33,34]. The main shapes of microplastics are fiber, flake, and line, which accounted for 39.5%, 28.4%, and 20.8%, respectively, and the main compositions are polypropylene, polyethylene terephthalate, and polyethylene, accounting for 41.0%, 25.1%, and 24.9%, respectively. Yellow and green microplastics were the most abundant, accounting for 21.9% and 19.6%, respectively, followed by black (16.5%), transparent (15.7%), white (10.7%), and red (10.4%).

### 3.3. Characterization of Seasonal Patterns of Microplastics

In spring, microplastics with a particle size of less than 1 mm were the most abundant, accounting for 41.0% of the total, followed by (1–2) mm, accounting for 39.3%. The main shapes of microplastics were flake and line, accounting for 72.8% and 16.2%, respectively; the main compositions were polypropylene and polyethylene, accounting for 71.0% and 20.0%, respectively; and yellow and green microplastics were the most numerous, accounting for 63.8% and 17.2%.

In summer, microplastics with particle size (1–2) mm were the most numerous, accounting for 34.7%. The main shapes of microplastics were fibrous and linear, accounting for 68.8% and 17.1%, respectively; the main ingredient was polyethylene terephthalate, accounting for 69.2%; and the largest number of green and black microplastics were found, accounting for 37.0% and 26.0%, respectively.

**Figure 5:**
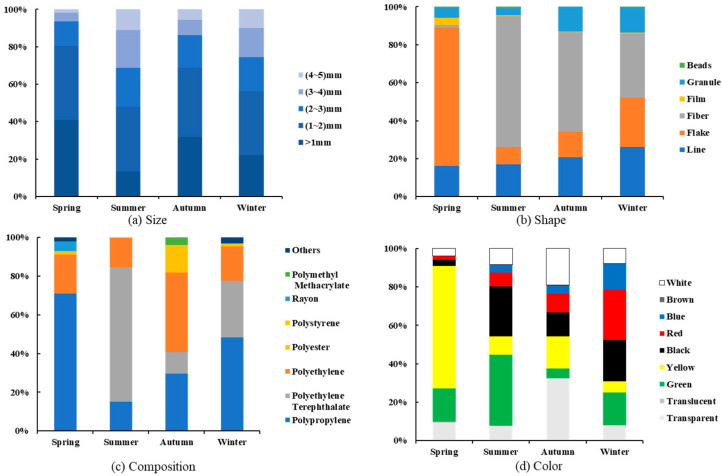
Physical and chemical characteristics of microplastics in different seasons.

In autumn, microplastics with particle size ranges of less than 1 mm and (1–2) mm accounted for the largest number of microplastics, 31.9% and 36.8%, respectively; the main shapes of microplastics were fiber and line, 52.4% and 20.7%, respectively; the main compositions of microplastics were polyethylene and polypropylene, 41.0% and 29.5%, respectively; and the largest number of transparent, white, yellow, and black microplastics were found, 32.6%, 32.6%, 16.0%, and 16.6%, respectively (Figure 5).

In winter, microplastics with particle size ranges of less than 1 mm and (1–2) mm accounted for the largest number of microplastics, with 22.1% and 34.2%, respectively; the main shapes of microplastics were fiber, line, and flake, with 33.7%, 26.1%, and 26.1%, respectively; and the main compositions were polyethylene, polyethylene terephthalate, and polypropylene, with 48.2%, 29.5%, and 17.6%, respectively; red, black, green, and blue microplastics were the most numerous, accounting for 25.9%, 21.7%, 17.1%, and 13.7%.

## 4. Discussion

### 4.1. Levels of Microplastic Pollution in the Sea off the Yangtze River Estuary

Although microplastic monitoring has long been carried out in both domestic and international countries, there is no uniform standard yet. Different scholars’ microplastic research methods are different, and the research differences are mainly the size of microplastic particles. For example, the particle sizes studied are (0.5~5) mm [26,27] and (0.05~3) mm [35], while the particle sizes of (0.33~5) mm [32,36,37,38,39,40,41] are the most studied. In order to assess the pollution level of microplastics in the sea area off the Yangtze River estuary, this paper compares with the results of foreign published studies on microplastics using nets of similar pore size.

The average abundance of microplastics in the waters off the Yangtze River estuary (0.17 items/m^3^) (0.00561 mg/m^3^) is in the same order of magnitude as that in the northwestern Mediterranean Sea [37], the Arctic Sea [38], and the Chukchi Sea [39] and is lower than that in the North Pacific Ocean [40], the California Sea [41,42], and the waters of the southeastern coast of South Korea [43], which suggests that the microplastics in the waters off the Yangtze River are at a moderately low level when compared to that of existing studies in foreign countries. Compared with the results of our coastal survey, it is in the same order of magnitude as the East China Sea Coastal [26], Bohai Sea [32], Jiangsu coastal area [44], Hangzhou Bay [45], Beibu Gulf [23], and Jinzhou Bay [46] and is higher than that of the South China Sea [47] and lower than that of Xiangshan Bay [48]. The level of microplastic pollution in the sea area off the Yangtze River estuary was at a moderately low level compared with domestic and international regions (Table 1).

### 4.2. Spatial and Temporal Distribution Factors of Microplastics off the Yangtze River Estuary

Microplastics were detected in different seasons at all stations except for station CJK5 in spring, where no microplastics were detected. Microplastics are commonly found in the sea area off the Yangtze River estuary, but their distribution is heterogeneous. The distribution of microplastics in seawater is affected by a variety of reasons, such as economic level, population density, circulation, wind, estuaries, harbors, and coastal sewage treatment plants in the countries along the ocean [50]. It was found that the abundance of microplastics at different stations in the sea area off the Yangtze River estuary varied greatly, and the abundance at nearshore stations was significantly higher than that in the farshore area. The overall spatial distribution of microplastics off the Yangtze River estuary showed a trend of higher abundance closer to the shore and lower abundance farther from the shore. In particular, the four-season abundance at station CJK1 was significantly higher than at the other stations, with an average abundance of 0.34 items/m^3^ (0.01122 mg/m^3^), while other stations yielded less than 0.2 items/m^3^ (0.0066 mg/m^3^). It is not difficult to see that the higher density of microplastics in the nearshore is mainly affected by the input from land sources, and the runoff of the Yangtze River has a decisive influence on the distribution of microplastics in the sea area outside the Yangtze River estuary. Preliminary analysis suggests that the distribution of microplastics in the sea area outside the Yangtze River estuary is mainly from riverine input in the nearshore, while riverine input is the main source of microplastics in the farshore, and human activities such as fishery fishing and shipping activities have aggravated the generation, aggregation, and diffusion of microplastics. 

The reasons for the high level of microplastics in the nearshore and low level in the farshore of the Yangtze River estuary are manifold: (1) The study area is close to Shanghai and Jiangsu; the regional economy is developed; regional commerce, tourism, aquaculture, shipping, ports, and other activities are prosperous; the population is large; the amount of plastic waste generated by human activities is huge; and a large number of land-based sources of plastic garbage for the sea microplastics provide a rich material base conditions. (2) The spatial distribution of microplastics is influenced by hydrology and river input. The Yangtze River is the richest river in China, accounting for about 36% of the total river runoff, and the runoff of the Yangtze River provides important power conditions for the diffusion of microplastics into the sea. Microplastics from land-based sources are more likely to migrate with the Yangtze River runoff, which becomes one of the main reasons for the higher microplastics in the nearshore sea. Plastics and microplastic particles enter the sea with river water, the seawater has a dilution effect on the concentration of microplastics, and the dilution effect is intensified with the increase in the transportation distance of the Yangtze River freshwater, which results in the farshore site being lower than the nearshore site.

**Figure 6:**
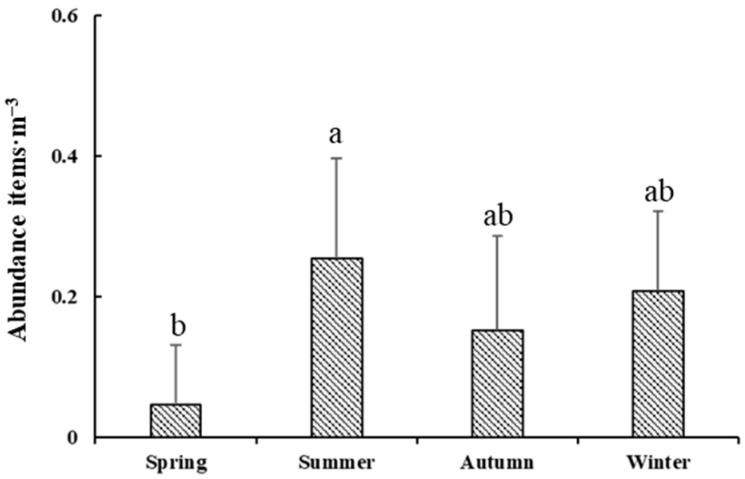
Comparison of microplastic abundance in different seasons. The letter indicated significant difference in Mann–Whitney U-test (*p* < 0.05).

The study area is located at the mouth of the Yangtze River, which is strongly influenced by the river, and there are obvious seasonal variations in microplastic abundance in surface waters (Figure 6). The abundance of microplastics in the surface waters off the Yangtze River estuary was significantly higher in summer than in spring, followed by winter and autumn. The high abundance of microplastics in summer is mainly due to the influence of the East Asian monsoon and the abundant precipitation, which leads to maximum runoff of the Yangtze River into the sea throughout the year.

Higher microplastic abundance was found in CJK5 and CJK4 at farshore stations in both fall and winter, which was significantly higher in winter than in autumn for several reasons. (1) Increased human activities: Frequent fishing activities occur after end of the fishing season in summer, and human activities influence the abundance and distribution of microplastics at sea to some extent, making higher concentrations in farshore areas possible. (2) Influence of the monsoon: Seasonal changes result in the summer prevailing southeasterly winds change to northerly winds, and the wind promotes the entry of plastic waste into the sea but also increases the content of microplastics in the ocean; on the other hand, the wind promotes the mixing of plastic debris in the ocean’s upper waters and vertical redistribution, which may lead to increased microplastic content. (3) The impact of runoff is weakening: The runoff volume of the Yangtze River into the sea gradually decreases after the autumn, and the fresh water flushing from the Yangtze River shifts from the northeast to the southeast, and the region is basically unaffected by the fresh water flushing from the Yangtze River in winter. (4) Cumulative effects: With the cumulative effects of human activities and wind, microplastic abundance is higher in the farshore region in winter than in autumn.

Lower levels of microplastics were found in the farshore region in spring, especially in CJK5, where no microplastics were detected. Due to seasonal changes, the runoff from the Yangtze River gradually increases, the direction of freshwater flushing shifts from the southeast to the northeast, and the prevailing wind shifts from northerly to southeasterly, so microplastics are less likely to accumulate in this area, and thus, the abundance is lower relative to fall and winter.

### 4.3. Analysis of Sources of Microplastic Pollution

**Figure 7:**
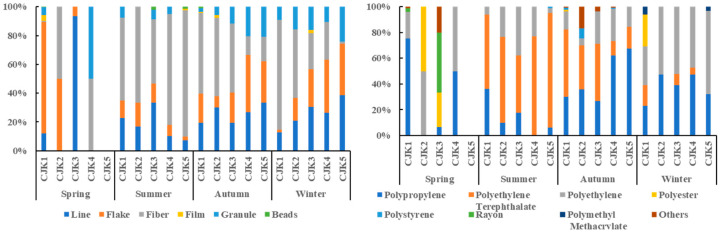
Types and components distribution of microplastics at sites.

The source of marine microplastics is a difficult point in the study of the microplastics under research. Shape and material are important ways to determine the source, and this study attempts to analyze the possible sources of surface seawater microplastics in the waters off the Yangtze River estuary based on the shape and composition of the plastics (see Figure 7). In the surface seawater microplastic samples, fibrous polyethylene terephthalate (24.2%), flaky polypropylene (21.7%), linear polypropylene (9.7%), and polyethylene (9.1%) were the main compositions of microplastics.

Polypropylene is a thermoplastic polymer obtained by polymerization of propylene monomers, with a high melting point, high strength, high heat resistance, high abrasion resistance, and low creep but also with good tensile and yield strength, rigidity, stress resistance, and electrical insulation and other excellent properties. Polypropylene is used in granulated polypropylene, DVD packaging materials, polypropylene materials, random polypropylene-modified asphalt waterproofing materials, washing machine materials, cast polypropylene film materials, automotive plastics materials, and woven products made with polypropylene. In daily life, it is widely used in the packaging of clothing, textiles, bread, and other goods and also used in cable production [51]. From the shape analysis of flake polypropylene, mainly from the broken plastic packaging bags, the color is mainly yellow; the greatest number was found in spring, and some of the samples had become discolored or even cracked due to prolonged immersion in seawater. Linear polypropylene was mainly derived from the breaking of harbor shipping and fishing cables and nets, and the colors were mainly yellow, white, and red.

Polyethylene is the most widely used of the polymer materials, mainly used to manufacture film, packaging materials, containers, pipes, monofilaments, wires, cables, daily necessities, etc., and can be used as a high-frequency insulating materials for television, radar, etc. Analyzed in terms of shape, linear polyethylene originates from the breakage of cables and nets in port shipping and fishing. The color is predominantly green. All of these plastics have various degrees of deterioration.

Polyethylene terephthalate (PET) is a thermoplastic obtained by polycondensation of terephthalic acid (TPA) and ethylene glycol (EG) or prepared by ester exchange of dimethyl terephthalate (DMT) and EG. PET has the advantages of non-toxicity, tastelessness, light weight, high transparency, and better mechanical properties, so it is widely used in the fields of food packaging, fibers, and electrical insulating materials and other fields [52]. PET fiber is the number one chemical fiber species, and its production accounts for more than 80% of the total production of chemical fibers [53]. Analysis of fibrous PET from the main samples showed that it is produced in the processing, use, and cleaning process of textiles and has a color variety, mainly black, red, transparent, and blue. Some studies have shown that the number of microplastics released per 3 g of fabric washing can be up to 1300–1500 roots [54].

The survey also found a small amount of polyester and rayon fibers, which came from the same source as PET fibers, and a small amount of polystyrene foam, which was analyzed to have originated from foam rafts used in nearshore aquaculture and fishing activities.

In spring, microplastics were mainly flake polypropylene (65.5%) and line polyethylene (8.6%). In summer, microplastics were mainly fiber polyethylene terephthalate (68.4%), line polypropylene (8.0%), and polyethylene (8.7%). In autumn, microplastics were predominantly fiber polyethylene (18.1%), polyester (14.3%), polyethylene terephthalate (9.3%), polypropylene (9.0%), line polyethylene (13.0%), and polypropylene (6.2%). In winter, it was mainly fiber polyethylene terephthalate (29.0%), line polypropylene 19.4%), and flake polypropylene (17.0%). Preliminary analysis deduces that plastic packaging materials enter the sea more in spring, followed by discarded cables from fisheries. Domestic water discharges such as sewage treatment plants and nearshore fishing activities are the main sources of microplastics in summer and autumn, and sources of microplastics are diverse in winter.

### 4.4. Ecological Risk Assessment of Microplastics

The ecological risk index method [55] not only takes into account the impacts of various pollutants on the environment in a particular depositional environment but also adequately reflects the combined effects of multiple pollutants in the environment to quantitatively classify the potential ecological risk level; thus, it is one of the important methods of assessing the potential ecological risk of sediments. Peng [56] improved on the basis of the traditional potential ecological risk index method to study the risk of microplastic contamination in the surface water of pump mining in the Yangtze River estuary. In this study, we attempted to use its method to assess and study the potential risk of microplastics in surface waters off the Yangtze River estuary.

The formula is as follows:

$$C_{f}^{i}=\frac{C^{i}}{C_{r}^{i}}\;\;T_{r}^{i}=\frac{P_{i}}{C^{i}}\times S_{i}\;\;E_{r}^{i}=T_{r}^{i}\times C_{f}^{i}\;\;RI=\sum_{i=1}^{n}E_{r}^{i}$$

$C_{f}^{i}$ is the pollution index of microplastics, *C^i^* is the measured concentration, $C_{r}^{i}$ is the standard reference value—here, we refer to the safe concentration of microplastics in surface waters of 6650 particles/m^3^ estimated by Everaert et al. [57]. We refer to Lithner et al. [58] to define the hazard index of microplastic polymers *S_i_*; *P_i_* is the concentration of specific microplastic polymer *i* concentration; $T_{r}^{i}$ is the ecotoxicity response factor (the sum of the product of the percentage of the total amount of plastic polymer *i* and the hazard index of that polymer); $E_{r}^{i}$ is the potential ecological risk index; *RI* is the ecological risk index of the microplastic polymers; *n* is the number of microplastic polymer species contained in the sample. We have listed the risk rank of polymers based on monomer toxicity in the Supplementary Material (Supplementary Material Table S1).

In terms of the pollution level of single-factor pollutants, the values of the abundance range of microplastics were non-detected to 0.44 items/m^3^ (0.0132 mg/m^3^), and the mean value was 0.17 items/m^3^ (0.00561 mg/m^3^), which was much lower than the safe concentration predicted by Everaert et al., indicating that the current status of microplastic pollution in the region is relatively light. In terms of potential ecological risk, the degree of microplastic contamination in the surface water network samples off the Yangtze River estuary was generally low, and the variation of the potential risk index ($E_{r}^{i}$) of each seasonal station ranged from 0 to 3.12 × 10^−4^, which indicated that the microplastic contamination status in the surface seawater off the Yangtze River estuary was relatively light (Table 2).

It has been found that microplastics are easier to distribute in the natural environment due to their small size compared to large plastics. At the same time, due to the large specific surface area of microplastics in the water column, they can easily combine with other substances to form larger combinations and accumulate in sediments. Therefore, the abundance of microplastics in sediments is much higher than in water bodies, and the environmental conditions of sediments are more complex, resulting in greater potential hazards of microplastics in sediments.

Zhang et al.’s [59] study of sediments in the Yangtze River estuary showed that sediments were the main sink for microplastics, which tended to be distributed vertically in the upper sediments and suspended phases after suspended sedimentation [60].The abundance of microplastics in the upper sediment was significantly higher than that in the lower sediment. Meanwhile, the distribution of microplastics with different densities in the vertical space was different; low-density microplastics float in the water usually easily float in the water, and those with higher densities more easily sink. Immobilized microplastics in sediments may be reactivated by disturbances at the water–sediment interface [61], allowing them to migrate upward into the overlying water. At the same time, biological effects can also cause vertical spatial changes in microplastics. After being accidentally ingested by an organism, the already-deposited microplastics may undergo changes through the digestive system of the organism, leading to changes in their spatial distribution. In the horizontal direction, the distribution of microplastic abundance in sediments is roughly the same as that in the water column, with high volume areas occurring mainly in shallow, nearshore waters. The reasons affecting the horizontal distribution of microplastics can be summarized as follows: First, due to the scouring effect of the river, microplastics in the center of the river are washed to the riverbank and deposited. Secondly, due to the lesser flow of shallow water close to the shore, microplastics are easily deposited. Third, low-density microplastics can easily move with the monsoon and be deposited on lakeshore sediments near estuaries. Fourth, human activities are intensive in coastal areas, and the spatial variability of microplastics is mainly influenced by the extent, path, and location of human activities [62]; thus, the abundance of microplastics is relatively great in shallow waters near the coast. In ecosystems, biological uptake is a key aspect of the microplastic transport process, and microplastics in sediments are taken up by a large number of aquatic organisms, including fish [63]. Microplastics have been reported to be widely detected in various organisms, such as fish [64,65]. Feng et al. [66] demonstrated that MPs can accumulate in the gills, intestines, and skin of fish; Su et al. [67] investigated microplastics concentrations in fish from the Yangtze River estuary and found that MPs accumulated in the gills and intestines but not in the liver or muscles of fish; similarly, MPs were found in the gills, stomach, and intestines of fish from the mangrove wetlands of Zhanjiang [68]; Song et al. [69] also found that the detection rate of microplastics in wild fish from Haizhou Bay, Lvshi, and the Yangtze River estuary reached 98%, with concentrations of 0.28 ± 0.23 items/g, and MP abundance was highest in the skin (1.40 ± 1.38 items/individual), followed by gills (1.23 ± 1.07 items/individual), intestine (0.90 ± 0.95 items/individual), and liver (0.72 ± 0.91 items/individual). The intake of microplastics by fish not only limits their growth but may also jeopardize human health through the food chain [70], causing significant damage.

## 5. Conclusions

(1) The abundance of microplastics in the surface seawater off the Yangtze River estuary is (0.17 ± 0.14) items/m^3^ (0.00561 ± 0.00462) mg/m^3^, which is at a moderately low level compared with other regions both domestic and abroad;
(2) In surface seawater, the number of microplastics with a particle size of (1~2) mm is the largest; their shapes are mainly fibrous, flaky, and linear; their compositions are mainly polypropylene, polyethylene terephthalate, and polyethylene; and their colors are varied, mainly yellow and green;
(3) Influenced by land-based inputs, hydrodynamics, and human activities, the spatial distribution of microplastics in the surface seawater off the Yangtze River estuary is uneven, with a high level near the shore and a low level far away from the shore; there are obvious seasonal variations, with a higher level of microplastic pollution in the summer; and the level of distribution is affected by the runoff significantly, with a higher level of pollution near the shore;
(4) Preliminary analysis suggests that marine shipping, fishing, and land-based sewage activities are important sources of microplastics in the waters off the Yangtze River estuary;
(5) The ecological risk index method can fully reflect the combined effects of multiple pollutants in the environment. The potential ecological risk of microplastics in the surface seawater off the Yangtze River estuary is small.

## Figures and Tables

**Table 1:** Comparison of microplastics between this study and other sampled areas.

Study Area	Net Mesh (μm)	Density (Items·m^−3^)	Study Period	Author
North Pacific Ocean circulation area	333	2.23	1999.8	Moore et al., 2001 [40]
Southern California coast	333	7.25	2000.10, 2001.1	Moore et al., 2002 [41]
Santa Monica Bay	333	3.92	2001.3	Lattin et al., 2004 [42]
Northwestern Mediterranean	333	0.116	2010.6–2010.9	Collignon et al., 2012 [37]
Arctic Sea	333	0.34 ± 0.31	2014.6	Lusher et al., 2015 [38]
Southeast coast of Korea	330	1.92–5.51	2012.5–2012.6, 2013.6–2013.7	Kang et al., 2015 [43]
Chukchi Sea	333	0.23 ± 0.07	2017.10	Mu et al., 2019 [39]
East China Sea coast	333	0.167 ± 0.138	2013.7–2013.8	Zhao et al., 2014 [26]
Bohai Sea	330	0.33 ± 0.34	2016.8	Zhang et al., 2017 [49]
South China Sea	333	0.045 ± 0.093	2017.4	Cai et al., 2018 [47]
Xiangshan Bay	333	8.91 ± 4.70	2017.10	Chen et al., 2018 [48]
Hangzhou Bay	330	0.14 ± 0.12	2019.10	Wang et al., 2020 [45]
Jinzhou Bay	330	0.65 ± 0.58	2016.10	Zhang et al., 2021 [46]
Beibu Gulf	300	0.1 ± 4.6	2018.10	Zhang et al., 2021 [23]
Jiangsu coastal area	200	3.1–3.5	2021.11	Xu et al., 2021 [44]
Off the Yangtze River estuary	330	0.17 ± 0.14	/	This study

**Table 2:** Risk-level criteria for microplastic pollution.

$\mathrm{Potential}\;\mathrm{Ecological}\;\mathrm{Risk}\;\mathrm{Factor}\;E_{r}^{i}$	˂10	10–100	100–1000	>1000
Ecological Risk Scale for Microplastic Pollution	I	II	III	IV

## Data Availability

Not applicable.

## Supplementary Materials

The following supporting information can be downloaded at: https://www.mdpi.com/article/10.3390/toxics11110889/s1, Table S1. Risk rank of polymers based on monomer toxicity.
