# Supplementary material for: Distribution Characteristics of Microplastics in Surface Seawater off the Yangtze River Estuary Section and Analysis of Ecological Risk Assessment
Source: Toxics. 2023 Oct 30;11(11):889. doi: 10.3390/toxics11110889 (PMC10674722; doi:10.3390/toxics11110889)
Supplement: Supplementary file 1 [file toxics-11-00889-s001.zip › toxics-2688232-supplementary.pdf]

Supplementary Materials

Distribution Characteristics of Microplastics in Surface Seawater off the Yangtze River Estuary Section and Analysis of Ecological Risk Assessment

Xiao Ji [1,2,+], Shuaishuai Yan [1,+] ,Yanlong He [1] Haisheng He [1] and Hanqi Liu [1,2,*]

[1] East China Sea Ecological Center,MNR Shanghai 201206, China
[2] Key Laboratory of Marine Ecological Monitoring and Restoration Technology, Ministry of Natural Resources, Shanghai 201206, China
* Correspondence: liuhanqi@ecs.mnr.gov.cn
\+ These authors contributed equally to this work.

Supplementary Material Table S1. Risk rank of polymers based on monomer toxicity

polymer	hazard index	Polymer monomers (wt.%)
polyether based flexible foam	13,844 (V)	Propylene oxide (58 wt.%)
Polyacrylonitrile (PAN)	11,521 (V)	Acrylonitrile (100 wt.%)
Polyvinyl chloride (PVC)	10,551 (V)	Vinyl chloride (50 wt.%)
Styrene acrylonitrile (SAN)	2788 (V)	Styrene (76 wt.%)
High-impact polystyrene (HIPS)	1628 (V)	Styrene (92 wt.%)
Polyacrylic acid (PAA)	230 (III)	Acrylic acid (100 wt.%)
Polystyrene (PS)	30 (II)	Styrene (100 wt.%)
